# Age at Menarche in Urban Girls Exposed to Lead in the Copper Basin, Poland

**DOI:** 10.3390/biology11040584

**Published:** 2022-04-12

**Authors:** Aleksandra Gomula, Natalia Nowak-Szczepanska, Anna Sebastjan, Sławomir M. Kozieł, Robert M. Malina, Zofia Ignasiak

**Affiliations:** 1 Department of Anthropology, Hirszfeld Institute of Immunology and Experimental Therapy, Polish Academy of Sciences, 53-114 Wroclaw, Poland; natalia.nowak-szczepanska@hirszfeld.pl (N.N.-S.); slawomir.koziel@hirszfeld.pl (S.M.K.); 2 Department of Biostructure, Wroclaw University of Health and Sport Sciences, 51-612 Wroclaw, Poland; anna.sebastjan@gmail.com (A.S.); zofia.ignasiak@awf.wroc.pl (Z.I.); 3 Department of Kinesiology and Health Education, University of Texas at Austin, Austin, TX 78712, USA; rmalina@1skyconnect.net

**Keywords:** lead, menarche, sexual maturation, toxicant

## Abstract

**Simple Summary:**

Lead is an environmental pollutant that negatively affects human growth, development and health. However, research into its effect on age at menarche (first menstruation), a proxy for maturation, is, to some extent, inconclusive. In this study, we identified that lower than currently acceptable blood lead level was related to later menarche. However, body weight and fatness had a moderating effect on this relationship, decreasing its significance.

**Abstract:**

Lead negatively affects human growth and development. In this research, we aimed to assess the effect of elevated blood lead level on age at menarche (AM), controlling for body mass index (BMI) and estimated fatness. The sample included 490 girls aged 7–16 examined in Polkowice town (Copper Basin, Poland) in 2008. Measurements included height, weight, skinfold thicknesses and estimated percentage of body fat. AM was assessed using the status quo method. Blood samples were taken for lead level assessment. Two groups were defined based on the median blood lead level for the total sample of children (3.7 µg/dL). Logistic regression models were used to assess the association between AM and independent variables. The results indicated that menarche in the higher blood lead level group was significantly later compared to the lower blood lead level group (*p* < 0.01). This relationship remained only marginally significant when BMI (*p* < 0.10), sum of skinfolds (*p* < 0.09) or percentage of fat (*p* < 0.08) were controlled. The results revealed that a lower blood lead level (3.7 µg/dL) than the currently acceptable threshold (5 µg/dL) is related to a later AM; however, this relationship is moderated by body fatness, which may decrease its significance.

## 1. Introduction

Industrial development has contributed to the improvement of living conditions, mainly since 20th century. However, environmental pollution associated with some industries has offset, to some extent, the benefits of socioeconomic advancement. Among environmental pollutants, lead (Pb) has received considerable attention and is recognized as an element that has potentially negative consequences for growth, maturation and health in general [1]. An elevated blood Pb level is associated with reduced linear growth [1,2,3,4], as well as disturbed neurodevelopmental and neuropsychological functions in children [5,6,7]. Some evidence also suggests an association of higher blood Pb level with lower values for indicators of nutritional and weight status [8].

Studies on the effect of blood Pb level on age at menarche (AM) generally suggest an association between elevated blood Pb levels and later age at menarche. In terms of prenatal or early postnatal periods, results revealed that higher early lead exposure could be related to later menarche [9,10]. However, regarding school-age girls, the results are, to some extent, inconsistent. For instance, Wu et al. [11] revealed that after adjustment for race/ethnicity, higher blood Pb level was significantly related to delayed menarche, whereas Selevan et al. [12] found that this relationship was ethnically specific and did not apply to non-Hispanic Caucasian American girls. In a study by Naicker et al. [13], non-Hispanic Caucasian girls were excluded due to their small number. Moreover, according to Slawinska et al. [14], there was no effect of blood Pb level on menarche in 1996, whereas in 2007, it was only marginally significant (*p* = 0.06). Lee et al. [15] revealed a positive relationship between blood Pb level and AM in South Korean girls, whereas Choi [16] found differing results, also in South Korea, where higher blood Pb level was related to earlier AM. Furthermore, although in the National Toxicology Program report [17], later maturation in girls was consistently noted at a higher blood Pb threshold level (>10 µg/dL), studies on the effects of lower Pb concentrations, particularly lower than the currently accepted threshold of 5 µg/dL, are relatively limited ([12,18]; divergent results of Choi [16] and Lee et al. [15]).

Therefore, the aim of this study was to assess the effect of blood Pb level, using a threshold lower than the currently accepted 5 µg/dL, on age at menarche in a sample of Polish school-age girls, controlling for relative body weight (BMI) and adiposity, which are variables related to maturation.

## 2. Materials and Methods

The study was conducted in 2008 in Polkowice town in the Legnica-Głogów Copper Basin in southwest Poland. Since the 1960s in this region, mines, plants and smelters related to the copper industry had been generating large amounts of industrial waste containing heavy metals. As a result, the air, soil and cereal crops in the Copper Basin have generally showed elevated lead concentrations relative to other regions of Poland [2]. However, since the early 1990s, intensive governmental interventions towards environmental protection have led to a profound reduction in harmful substance emissions, which has significantly contributed to regional air quality improvement [1,19]. As a result, since 2004, the average annual Pb level in the Copper Basin has not exceeded the permissible average level for Poland, i.e., 0.5 µg/m^3^ [20]. The average annual concentration of Pb in Polkowice in 2007 was 0.032 µg/m^3^, which was 6% of the permissible average annual Pb level [21].

The study was approved by the Committee for Scientific Research Ethics at the University of Physical Education in Wrocław (8 March 2005) and the school authorities in Polkowice. Both the girls and their parents or legal guardians gave their informed consent to participate in the study.

The sample included 490 girls, 7–16 years of age, who were attending several schools in Polkowice in 2008. Age at menarche was assessed using the status quo method. All girls provided information on their menarcheal status, i.e., whether menarche had already occurred (yes) or not occurred (no) at the time of examination. Height, weight and three skinfolds were measured by qualified staff from the Department of Biostructure of the University of Physical Education in Wroclaw. All measurements were taken in the morning in a special room; the girls were in light sportswear without shoes. Height was measured using an anthropometer to the nearest 0.1 cm, and body mass was measured to the nearest 0.1 kg using a medical scale [2]. Skinfold thicknesses were measured with a GPM caliper to the nearest 0.2 mm at three sites: triceps, subscapular and abdomen. The sum of the three skinfolds was used as an indicator of subcutaneous adiposity. The body mass index (BMI = weight/height^2^, kg/m^2^) was calculated.

Percentage of body fat (%fat) was estimated by near infrared interactance using a Futrex 5000A/ZL apparatus (Futurex Inc., Hagerstown, MD, USA). The validity of infrared interactance estimates of body composition in adolescents has been confirmed by research (e.g., [22]). The optical head of the unit contains four infrared light-emitting diodes with two wavelengths of 740 and 750 nm. The silicone detector measures the intensity of the reflected light at the measurement site, the center of the biceps brachii muscle as the midpoint of the distance between the acromion process and the cubital fossa (fossa cubiti) of the elbow. The midpoint was measured with a ruler. Age, sex, height and weight of the individual were entered into the internal software of the Futrex unit to derive estimates of absolute (kg) and relative (%) body fat based on the principles of light absorption and reflection [23].

Blood samples were taken by qualified personnel of the Foundation for Children in the Copper Basin. The samples were taken with an intravenous tube and a blood pipette and were subsequently tested for Pb levels by atomic absorption spectrometry in a Hitachi Z-8200 graphite-tube furnace with Zeeman background correction using standard laboratory procedures. Analyses were conducted at the accredited Heavy Metals Toxicology Laboratory (Legnica, Poland) using appropriate reference standards (Nycomed, Sweden). The minimal detectable level of Pb was <0.1 µg/dL. In this sample, there were no cases with levels below the detection limit. Laboratory procedures in the Heavy Metals Toxicology Laboratory at the Foundation for Children from the Copper Basin were regularly subjected to quality control [24].

The study sample was divided into the two groups based on the median blood Pb level for the total sample: <3.7 µg/dL (group 1) and ≥3.7 µg/dL (group 2) (Table 1). Probit analysis, which is a type of regression, was used to estimate the median AM for the total sample and the two blood Pb level groups. As the probit analysis assumes the normality of the distribution, it was justified to assume that the median equals the mean, and the standard deviation was calculated. The difference between estimated AM in the two groups was calculated using a test to assess the significance of the difference between the two means with known standard deviations and the number of girls in each group.

Because the data for age, height, weight, BMI, %fat, each skinfold and the sum of skinfolds did not meet the assumption of normality of the distribution (Kolmogorov-Smirnov and Lilliefors tests, *p* < 0.01), the differences between the two blood Pb level groups for these variables were calculated with non-parametric Mann–Whitney U tests.

Logistic regression was conducted to assess the association of the dependent dichotomous variables (menarcheal status: pre- or post-menarcheal) with independent variables (age, blood Pb level, BMI, %fat and sum of skinfolds). Due to the high collinearity of BMI, %fat and sum of skinfolds, three separate analyses for these variables were conducted. All calculations were performed with StatDirect 10.0 (StatsDirect Ltd., Wirral, UK) and Statistica 13.1 (TIBCO Software Inc., Palo Alto, CA, USA).

## 3. Results

**Figure 1:**
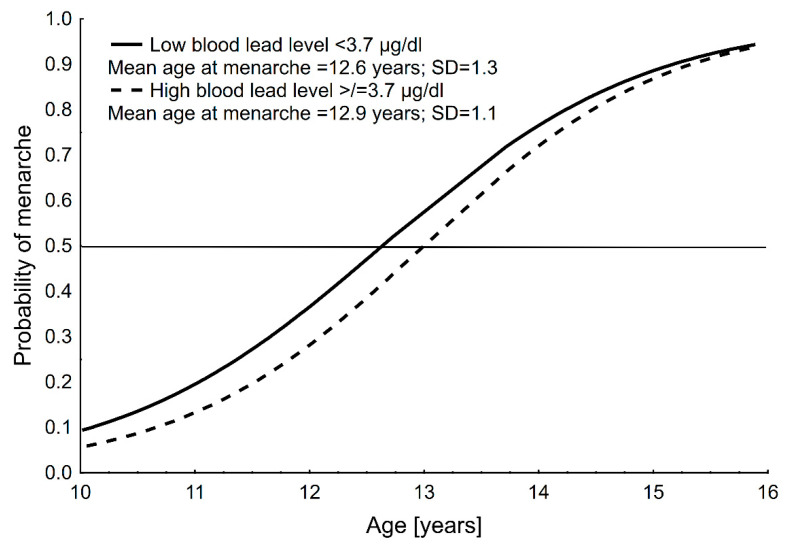
Distribution of the probability of menarche with age in the two blood lead level groups of girls in Polkowice in 2008. Age at menarche was assessed using the status quo method and calculated with probit analysis.

Mean age at menarche was 12.7 ± 1.2 years (CI: 12.5–12.9) for the total sample, 12.6 ± 1.3 years (CI: 12.3–12.9) for group 1 (<3.7 µg/dL) and 12.9 ± 1.1 years (CI: 12.8–13.3) for group 2 (≥3.7 µg/dL); menarche in the higher blood Pb level group was significantly later compared to lower blood Pb level group (*p* < 0.01). Graphical results of the probit analyses for AM in the two blood Pb level groups are presented in Figure 1.

Descriptive statistics for age, height, weight, BMI, %fat, particular skinfolds and the sum of skinfolds for the total sample and the two blood Pb level groups are presented in Table 2. The difference between the two blood Pb level groups in height was marginally significant (*p* = 0.05); the differences in all other variables between the two groups were not statistically significant (*p* > 0.05).

Results of the three logistic regression models are summarized in Table 3. In the three separate analyses, a significant effect of age (*p* < 0.0001) and (a) the BMI (*p* < 0.01), (b) %fat (*p* < 0.05) and (c) sum of skinfolds (*p* < 0.05) on AM is apparent, whereas the effect on blood Pb level is marginally significant: (a) BMI model (*p* = 0.099), (b) %fat model (*p* = 0.08) and (c) sum of skinfolds model (*p* = 0.09). The results suggest that as the BMI, %fat or sum of skinfolds increases (controlling for age), the probability of menarche also increases. At the same time, a higher blood Pb level is associated with later menarche, although its effect is only marginally significant.

## 4. Discussion

Most of previous studies have focused mainly on the neurotoxicity of lead that results in impaired neurobehavioral development or linear growth, whereas those concerning age at menarche are few (especially regarding the effect of lower than currently accepted Pb threshold) and provided incomprehensive results, particularly in terms of non-Hispanic Caucasian girls (see Introduction). Our findings contribute to the previous research on the effect of lead on human development regarding its adverse consequences in terms of maturation and broadens knowledge on this issue. This study, conducted on nearly 500 Polish schoolgirls who were under long-term exposure to elevated levels of lead, revealed that girls with a blood Pb level ≥ 3.7 µg/dL had their menarche significantly later compared to girls with a blood Pb level < 3.7 µg/dL. However, the relationship between menarcheal status and blood Pb level remained only marginally significant after adjustment for relative body weight or body fatness. Thus, relative body weight or adiposity seem to have, to some extent, a more pronounced effect on menarche, at least when the blood Pb level is relatively low.

Although the currently acceptable blood Pb level is 5 µg/dL [25], it is possible that long-term exposure to lower levels of blood Pb may have consequences for growth and maturation. Some researchers have already indicated that there is no safe level of exposure to lead for either children or adults [18,26,27], and therefore, any “effect threshold” for blood Pb level would be arbitrary and unwarranted [28]. Moreover, a growing body of evidence suggests that the long-term effects of lead exposure may be irreversible [27]; there is little evidence that neurodevelopmental deficits associated with early lead exposure resolve over time [26], its adverse effects often persist from early childhood to adulthood [26], and increase the risk of adult cardiovascular disease mortality [29].

Our results correspond, in some respects, with previous research conducted in the Copper Basin on rural girls who also came from the families of mine and factory workers in the copper industry [14]. In this population, the median age at menarche declined from 13.4 years in 1995 to 13.2 years in 2001 and 2004, and to 12.8 years in 2007. Blood Pb levels in 1995 and 2007 also declined over this interval, from 6.57 ± 0.13 µg/dL (range 2.00–33.90) to 4.24 ± 0.14 µg/dL (range 2.00–11.00). In 2007, controlling for height and BMI, the probability of attaining menarche decreased with an increased blood Pb level, although the effect of blood Pb level, similarly to in urban girls, was marginally significant (*p* = 0.06) (in 1995, this effect was insignificant) [14]. The mean blood Pb level of girls from the villages in 2007 (4.24 µg/dL) was higher than that of girls from Polkowice in 2008 (3.72 µg/dL), whereas ages at menarche in 2007 (villages) and 2008 (present study) were similar: 12.8 ± 1.2 years (CI: 12.6–13.0) and 12.7 ± 1.2 years (CI: 12.5–12.9), respectively. Perhaps the similarly high socioeconomic status of the families living in the Copper Basin (both rural and urban; see below) was related to the similar age at menarche of girls from the urban and rural areas of this region, and similar marginally significant effect of lead on AM when controlling for body size.

Age at menarche has significantly declined in recent decades in Poland as in other developed countries. In 2012, the mean age at menarche for large cross-sectional Polish sample was 12.9 years [30]; for towns (similar to the size of Polkowice) and villages, it was 13.1 years, and for cities, it was 12.7 years [30,31]. Note that despite higher Pb pollution in the Copper Basin (compared to other parts of the country), age at menarche in the Polkowice sample in 2008 was lower compared to the national average and the average for small towns in 2012 [30]. Additionally, in terms of villages in the Copper Basin, mean age at menarche in 2007 was 12.8 years [14], which was also lower than the national average and that of Polish towns and villages [30]. Because earlier menarche generally occurs among girls living under better socioeconomic circumstances, the earlier ages at menarche among girls living in the Copper Basin (compared to those from other Polish regions) may reflect better living conditions and the higher socioeconomic status of the region. Intensive investment and socioeconomic development in Polkowice started in the 1990s. The program involved cooperation among the local government, businesses and residents aimed at improving the living conditions in the community. The “growth machine” and “entertainment machine” policy models contributed to the establishment of a dynamically developing and innovative town. In 1999, the municipality of Polkowice was one of the richest in Poland [32,33]. It seems plausible that the favorable living conditions in this region compensated, to some extent, for the negative environmental effects of relatively low lead levels on age at menarche.

As mentioned in the Introduction, in terms of higher blood Pb level thresholds (compared to our study) in different countries, results are rather conclusive. In South Africa, where blood Pb levels are generally higher than in resource-rich countries, higher blood Pb levels (mean = 4.9 μg/dL) were associated with significant delays in the onset of puberty and later menarche [13]. Additionally, in a national sample of U.S. girls, a blood Pb level ≥5.0 µg/dL was associated with delayed onset of breast development and with later menarche [11]. However, when considering blood Pb levels similar to those in our research, results are less conclusive. In a study of U.S. girls, a blood lead level ≥3.0 µg/dL was associated with a delay in age at menarche in non-Hispanic African American girls but not in non-Hispanic Caucasian American and Mexican American girls [12]. An analysis of multichemical exposure among Akwesasne Mohawk Nation adolescent girls also suggested that menarche might be sensitive to relatively low Pb levels. In this sample, the predicted age at menarche for girls with blood Pb levels above the median (1.2 µg/dL) was later than that for girls with blood Pb levels below the median [18]. Research conducted in South Korea provided contradictory results. According to Lee et al. [15], a higher blood Pb level (mean = 1.02 µg/dL) correlated with later menarche, whereas Choi [16] revealed an opposite relation: higher Pb level (mean = 1.15 ± 0.04 μg/dL) was related to earlier menarche. Note that only Selevan et al. [12] demonstrated the relation between elevated blood Pb level and AM (with a threshold lower than 5 µg/dL) in non-Hispanic Caucasian girls, and this relationship was insignificant. Therefore, our research contributes to the current knowledge on these issues. However, further studies are still needed.

It has been suggested that changes in the endocrine system associated with Pb exposure may contribute to the delayed onset of pubertal maturation and age at menarche (e.g., [13,18,34]). Lead is a reproductive toxicant that affects the endocrine system and belongs to the endocrine-disrupting chemicals (EDCs) [35,36]. Lead can activate the estrogen receptor and initiate transcription of estrogen-activated genes [35]. Based on animal models, exposure to Pb resulted in an alteration in the affinity of estrogen and luteinizing hormone (LH) receptors [37,38,39] and appeared to act on the hypothalamic–pituitary axis, altering the release of LH needed for estradiol synthesis [40]. Furthermore, a reduction in plasma levels of insulin-like growth factor (IGF-1), LH and estradiol [40,41,42], as well as delayed puberty were found in the offspring of lead-exposed rats [43]. Mechanisms responsible for lead acting as an EDC might be found in the reduction in expression of the steroidogenic acute regulatory protein (StAR) [40] and in the inhibition of LH secretion [40,42].

However, this research has some limitations. In this study, we used a cross-sectional design. Therefore, it was not possible to obtain the exact AM of individuals, as is the case with the most reliable prospective (longitudinal) method. Instead, AM was assessed using the status quo method. Nevertheless, this limitation is rather unlikely to affect the results obtained, as the status quo and prospective methods yielded comparable results for ages at menarche in a sample (e.g., [44]). The lack of socioeconomic information about the families is another limitation, especially in the context of AM. Menarche is highly sensitive to living conditions and socioeconomic status of the family (e.g., [45]). In the present study, only 30% of respondents (for whom menarcheal status was available) provided information on socioeconomic status. Such a low response rate made it problematic to include this variable in the analyses. Nevertheless, the population of Polkowice is one of the richest in the country and relatively homogeneous in terms of living conditions and high socioeconomic status (see: [8]).

## 5. Conclusions

A lower (3.7 µg/dL) than currently acceptable (5 µg/dL) blood Pb level was related to later age at menarche. However, the association was only marginally significant when relative weight or body fatness were statistically controlled, implying their moderating effect on the influence of lead on age at menarche.

## Figures and Tables

**Table 1:** Means, medians, standard deviations (SD) and range of blood lead levels (µg/dL) in the total sample of girls and in the two blood lead level groups.

	N	Mean	Median	SD	Min	Max
Total	490	3.6	3.6	0.92	2.00	7.80
<3.7 µg/dL	276	2.9	2.8	0.47	2.00	3.60
≥3.7 µg/dL	214	4.4	4.3	0.58	3.70	7.80

**Table 2:** Anthropometric characteristics of the total sample of girls and of the groups of blood Pb levels, as well as the results of Mann–Whitney U tests (comparing the two blood Pb level groups).

	Total Sample				Group 1 (<3.7 µg/dL)				Group 2 (≥3.7 µg/dL)				
	N	Mean	Median	SD	N	Mean	Median	SD	N	Mean	Median	SD	Z
Age [years]	490	11.4	11.4	2.5	276	11.6	11.6	2.4	214	11.2	11.2	2.7	−0.96
Height [cm]	490	147.6	148.6	14.6	276	148.8	150.5	13.9	214	146.0	146.6	15.3	−1.93 *
Weight [kg]	490	41.7	40.9	13.8	276	42.7	41.3	14.2	214	40.5	40.1	13.3	−1.42
BMI [kg/m^2^]	490	18.6	17.8	3.6	276	18.8	17.8	3.8	214	18.5	17.7	3.2	−0.33
Fat [%]	476	19.0	16.7	8.5	269	19.2	16.9	8.9	207	19.7	16.5	8.0	−0.73
TSF [mm]	490	13.1	12.4	4.5	276	13.3	12.2	4.8	214	12.9	12.4	4.2	−0.34
SSF [mm]	490	9.6	8.2	4.8	276	9.8	8.2	5.2	214	9.2	8.2	4.1	−0.70
ASF [mm]	490	17.3	16.0	8.4	276	17.4	16.0	8.4	214	17.2	16.1	8.4	−0.37
Sum SF [mm]	490	40.0	36.4	16.6	276	40.6	36.2	17.3	214	39.3	36.5	15.7	−0.42

* *p* = 0.05; TSF, tricep skinfold; SSF, subscapular skinfold; ASF, abdominal skinfold; Sum SF; sum of skinfolds.

**Table 3:** Results of three logistic regression models with menarcheal status (no = 0, yes = 1) as the dependent variable and age, blood lead level (binary variable: low and high), BMI (a), %fat (b) and sum of skinfolds (c) as independent variables.

		OR	−95% CI	+95% CI	Wald’s *χ*^2^
(a)	Age	4.56	3.31	6.29	85.96 ****
	BMI	1.20	1.10	1.34	10.55 **
	Blood Pb level	0.54	0.26	1.13	2.72 ^a^
(b)	Age	4.13	2.93	5.82	66.07 ****
	% fat	1.05	1.01	1.10	4.76 *
	Blood Pb level	0.52	0.25	1.08	3.14 ^b^
(c)	Age	4.67	3.39	6.43	89.22 ****
	Sum of skinfolds	1.02	1.00	1.05	4.44 *
	Blood Pb level	0.53	0.26	1.10	2.89 ^c^

^a^*p* = 0.099; ^b^ *p* = 0.077; ^c^ *p* = 0.089; * *p* < 0.05; ** *p* < 0.01; **** *p* < 0.0001.

## Data Availability

Restrictions apply to the availability of these data. Data were obtained from Wroclaw University of Health and Sport Sciences and are available from the authors with the permission of Wroclaw University of Health and Sport Sciences.
